# Influence of Surfacing Fe-Based Alloy Layers on Wire Arc Additive Manufactured Ni-Based Superalloys Material on Its Microstructure and Wear Properties

**DOI:** 10.3390/ma15176020

**Published:** 2022-08-31

**Authors:** Yingyan Yu, Zhiyuan Qu, Jiansheng Zhang, Jie Zhou

**Affiliations:** 1 Chongqing Key Laboratory of Advanced Mold Intelligent Manufacturing, College of Materials Science and Engineering, Chongqing University, Chongqing 400044, China; 2 Chongqing Jiepin Technology Co., Ltd., Chongqing 400044, China

**Keywords:** microstructure, wear behavior, Ni-based superalloy, wire arc additive forming

## Abstract

Wire arc additively manufactured (WAAM) Ni-based materials have good properties but are costly and hard to cut, leading to difficulties in machining after welding and wasting the materials. To overcome these shortcomings, this work proposes a method of surfacing Fe-based alloy layers on WAAM Ni-based material. The effect of this method on the microstructure and wear properties of WAAM Ni-based materials is discussed. In this work, a Fe-based alloy (JX103) was welded as the last layers of the WAAM Ni-based superalloy (JX201) material. The hardness, microstructure, and wear behavior of the material with different residual Fe-based materials were tested and analyzed. Our results indicate that the surface hardness was smoothly increased from HV350 to HV400 by overlaying Fe-based alloy layers. Microstructure analysis shows that γ-Fe gradually disappears, and the carbide form changes from WAAM Ni-based superalloys to Fe-based alloys. In the fusion boundary, the occurrence of cellular dendritic growth, a type -Ⅱ boundary, and low dilution indicate good crack resistance and good connection performance between these two materials. The wear test showed that the wear resistance of JX201 was decreased by changing the last layer to JX103. However, as the residual thickness of JX103 decreased, the influence gradually reduced. Meanwhile, the wear mechanism changed from severe abrasive and adhesive wear to light abrasive wear. When the thickness is less than 0.5 mm, the wear weight per minute is at the same level as the sample without JX103.

## 1. Introduction

Hot forming tools are often associated with low service life and high costs [1,2]. The commonly used methods to improve tool life, such as surface treatments and laser cladding, usually produce thin protective surface layers on the die surface [3]. The protective effects are limited, and the equipment is dimensionally limited, limiting the economic cost reduction. Therefore, a series of methods for getting thicker hard surface layers and lower costs have been developed, such as laser deposition, composite surface treatment, and dissimilar metal additively manufacture [4].

Wire arc additively manufactured (WAAM) material is one of the most economical methods. Shen et al. [5,6] proposed a bimetal-layer surfacing technology by which a hot forging die is designed as the combination of a casting-steel base and two surfacing welded working layers. Since the surface layer obtained by WAAM can even reach a thickness of tens of millimeters, it can withstand the thermal and mechanical loads during forging with a proper thickness. The other part of the forging die can be made with cheaper materials, such as casting steel. According to statistics, the die costs were reduced by 30%, while the manufacturing cycle of the mold was reduced by 10%, and the materials were saved by 5% by adopting this method [7]. Furthermore, Xia et al. [8] studied the materials of the WAAM working layers and found that the tool life can be highly improved using Ni-based or Co-based superalloy layers on the die surface. Meanwhile, die crack can be alleviated by surfacing a transition layer between the surface layer and the die base. WAAM has considerable advantages in die manufacturing because of its high deposition efficiency, high wire utilization, short manufacturing cycle, high equipment flexibility, and low production cost [9]. WAAM dissimilar material can provide different properties for areas with different needs, which is of great significance to manufacturing dies and even large-scale high-performance parts. 

Many researchers studied WAAM dissimilar materials, mainly focusing on the mechanical and corrosion properties of different types of stainless steel, Ni-based superalloy, Co-based superalloy, and so on [10,11,12,13,14,15]. WAAM Ni-based superalloy has been closely studied since its high stability at high temperatures and good mechanical properties [16,17]. However, Ni-based superalloy is costly and hard to cut, which leads to difficulties in the machining after welding and wasting of the cut materials. In addition, the large heat input of WAAM leads to dimensional control being one of the challenges of WAAM [17]. The last welded layer usually functions as welding annealing layers and will be partly machined out afterward. Therefore, this work proposes a method of surfacing Fe-based alloy layers on WAAM Ni-based material as the last layers to improve machinability and reduce material waste. The hardness, microstructure, and wear behavior of the WAAM Ni-based material with different residual Fe-based materials were tested and analyzed. 

## 2. Materials and Methods

### 2.1. Materials and Wire Arc Additive Forming

A 100 mm × 100 mm block of medium carbon steel, C45, was used as the die base material, on which a self-developed Ni-based superalloy JX201 (similar standard: AWS A5.14: ERNiCR-3) was welded additively using wire arc welding. Six layers of JX201 were welded, and the total thickness reached 20 mm. Then 2 layers of Fe-based alloy JX103 (similar to RMD248) were welded on the top as the last layers and the welding annealing layers. The additive manufacturing process was carried out by gas metal arc welding (GMAW) using a ZX5-250 type DC welder. The welding parameters were as follows: mean welding current, 220 A; mean arc voltage, 28 V; electrode traveling speed, 11,000 mm/min; and the interpass temperature was kept at 300–320 °C. The chemical compositions of the mentioned three materials are shown in Table 1. After welding, the block sample was heated to 550 °C and kept for 1 h, then cooled in the furnace.

### 2.2. Microstructural and Properties Analysis

**Figure 1:**
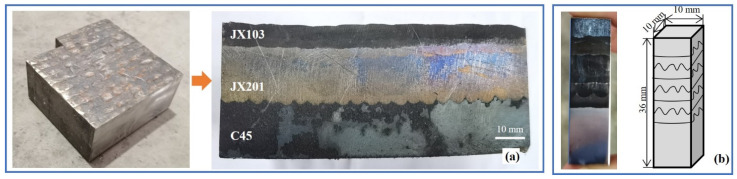
(**a**) Additively manufactured block sample (100 × 100 × 36 mm^3^). (**b**) The sample for microstructural inspection (10 × 10 × 36 mm^3^).

The block sample after the post-weld heat treatment is shown in Figure 1a. After the heat treatment, the hardness of JX103 is approximately 500 HV, while the hardness of JX201 is around 300 HV. The Vickers microhardness of each layer was tested by an HVS-1000z hardness tester with a load of 1000 g and a dwell time of 10 s in accordance with ISO 6507-1:2005. To find out the microstructure influence of the last two layers of Fe-based superalloy on the Ni-based superalloy, a sample with a width of 10 mm was taken on the cross-section, as shown in Figure 1b. The sample was etched in HNO3:HCl solution in a volume ratio of 2:1 and inspected with an OLYMPUSGX-41 type microscope. The alloys phases were identified using a Rigaku D/max2500PC X-ray diffractometer with CuKα radiation in the 2θ range of 10°–100°. The microstructural changes and elements’ distribution perpendicular to the welding plane were characterized by Olympus optical microscopy (OM) and scanning electron microscope (SEM) equipped with the Energy Dispersive Spectrometer. An Energy Dispersive X-ray Spectrometer (EDS) was used for Semi-quantitative chemical analyses of the phases present in the welded layers. Based on the weight percentage of Fe detected by the EDS, the volumetric dilution (Dv) of the two fusion boundaries was calculated to compare their connection performance.

### 2.3. Sliding Wear Test

**Figure 2:**
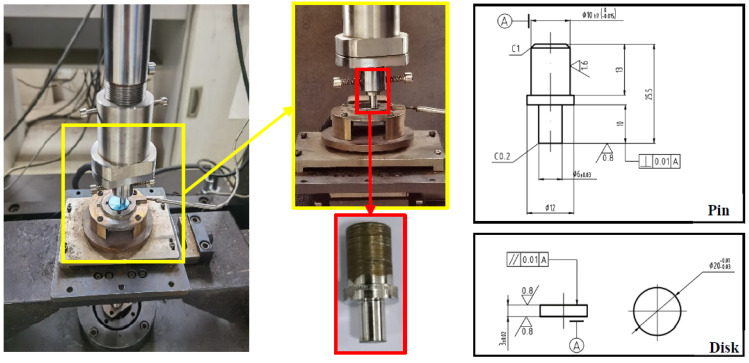
High-speed reciprocating friction and wear test machine (tribometer) and wear sample dimensions.

**Figure 3:**
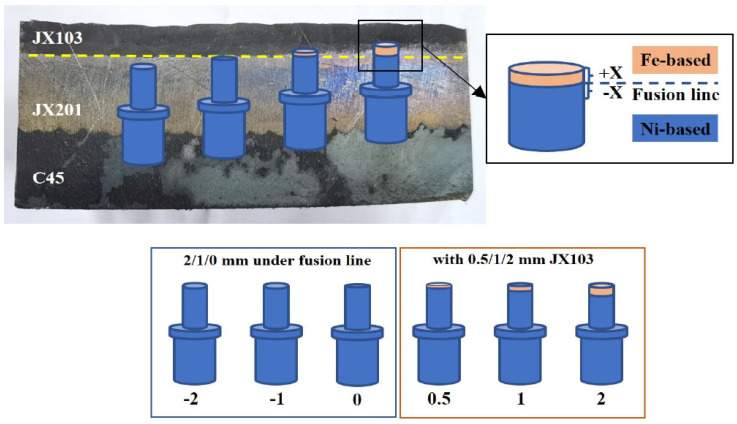
Pin samples and the sampling positions (X: distance between pin top and the fusion line).

Sliding wear tests were carried out on a high-temperature pin-on-disk tribometer based on ASTM G99-17 standard with the test material in contact with a disk. The disk is made of C45 steel, which is a commonly used material for forging parts. The pin and disk samples were machined and rigidly held, as shown in Figure 2. To explore the wear behavior difference caused by changing the material of the last surfacing layers, the pin samples were taken from different positions, as shown in Figure 3. These positions were taken according to the distance (X mm) between the sample’s top surface and the fusion line of JX103 and JX201. When the sample’s top surface was inside the JX103 layers, the sample was named +X. In contrast, the sample was named -X when its top surface was inside the JX201 layers. By changing positions, 6 series of pin samples (2, 1, 0.5, 0, 1, 2) were obtained with a different margin of JX103. The sliding wear tests were carried out without lubrication at a sliding speed of 100 mm/s under an applied load of 100 N at 300 °C for 10, 20, 30, 60, and 90 min, respectively.

## 3. Results and Discussion

### 3.1. Microhardness

**Figure 4:**
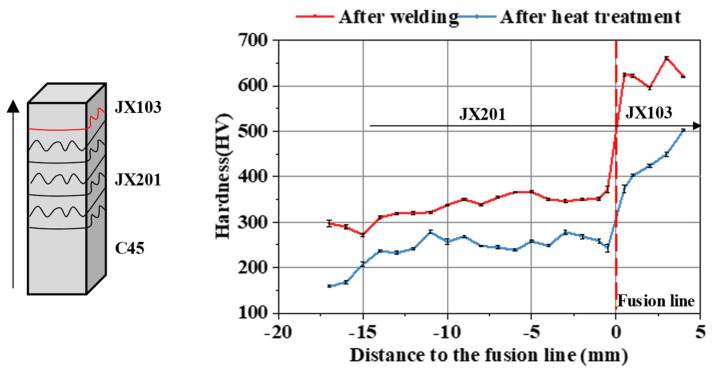
Microhardness distribution difference after welding and heat treatment (along the building direction).

The microhardness distributions after welding and heat treatment were compared in the direction perpendicular to the welding plane, as shown in Figure 4. After welding, the mean hardness of JX103 and JX201 was HV620 and HV350, respectively. After heat treatment, the hardness of JX103 decreased to HV400-500, while the hardness of JX201 decreased to around HV300. Since the tempering stability of JX201 is better than JX103, the hardness difference on the two sides of the fusion line is significantly reduced by the heat treatment. After tempering, in JX103, the second phase precipitates and aggregate, the desolubilization, aggregation, and growth of carbides are also obvious, and the dislocation density in the matrix is greatly reduced, which lead to a decrease in hardness. By changing the last surfacing layers from JX201 to JX103, the surface hardness is increased; meanwhile, the hardness transition is smooth [18].

### 3.2. Microstructure and Phase Characterization

**Figure 5:**
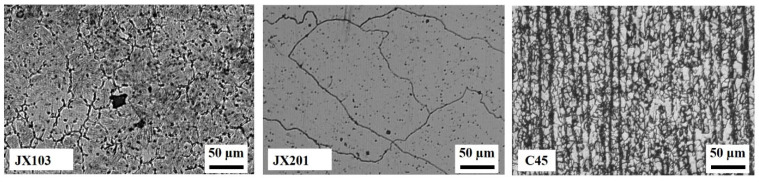
Microstructures of JX103 (Fe-based), JX201 (Ni-based), and C45 (base material).

Figure 5 shows the welded material’s microstructures of the base material C45 and the welded material of JX103 and JX201. It can be seen that the welded microstructures of the two materials are quite different. JX103, as a Fe-based superalloy, has smaller grains (4–5 times smaller than JX201), and many lath martensites are distributed inside the grains. While JX201, the Ni-based superalloy, has larger grains, and within the grain are dendrites. Therefore, in terms of mechanical properties, the hardness and strength of the welded material of JX103 are higher than that of JX201. 

**Figure 6:**
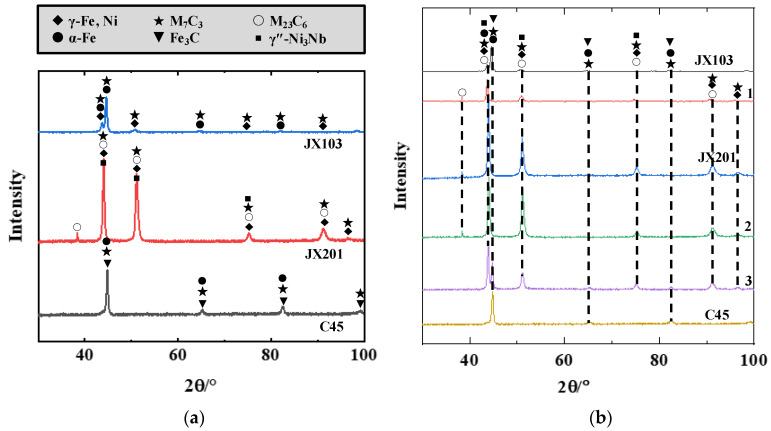
XRD analysis result of the welded materials at six different positions (along the building direction). (**a**) XRD results of C45 and welded materials of JX201 and JX103; (**b**) XRD results of the three materials and the fusion boundaries in between.

Together with the XRD analysis result shown in Figure 6a, the phase of each welded material can be identified. The microstructure of the JX103 consists of cellular austenite (γ-Fe) along with lath martensite and carbides inside. The microstructure of JX201 consists mainly of the γ phase, in which carbides and γ″-Ni3Nb phase are dispersed. Barrick et al. presented a similar observation for nickel-rich ferritic steel [19]. According to the XRD analysis results, the carbides can be identified as M7C3 and M23C6. The C45 thick plate, as the welding substrate, retains obvious traces of rolling. Undissolved ferrite was tempered, but the carbides sorbite and retained austenite are its main phases.

**Figure 7:**
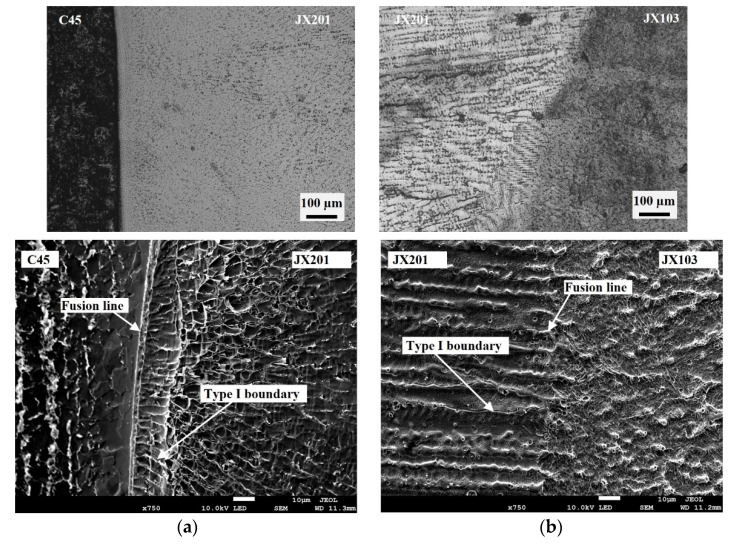
Microstructures of the two fusion boundaries: (**a**) Fusion boundary of JX201 and C45; (**b**) Fusion boundary of JX103 and JX201.

Figure 6b shows the XRD analysis result of the three welded materials in between and near the fusion boundary. The XRD patterns indicate that along the direction from the bottom welded layer, where JX201 was welded on a C45 thick plate, to the surface welded layer, the γ phase disappears gradually. Meanwhile, the form of carbides changed. Figure 7 shows the microstructures of the two fusion boundaries. The fusion boundary of JX201 and C45, as shown in Figure 7a, includes a fusion line and a transition zone. Thus, it was referred to as the widened fusion boundary [20]. In this fusion boundary, the bottom of the welded layer, which contacts the C45 substrate, mainly consists of cellular structures. On these cellular structures, obvious cellular-dendritic growth solidifications were observed. The oriented microstructure resulted from the gains’ higher growth rates blocking their neighbors. Type-I boundaries (perpendicular to the transition zone) were observed in this region [21,22]. Figure 7b shows the fusion boundary of JX103 and JX201. Cellular dendritic growth was observed from the fusion boundary. The fusion boundary was relatively sharper than the fusion boundary of C45 and JX201. Moreover, only type-I boundaries but no type-II boundaries were observed, which indicates the crack resistance of this fusion boundary is relatively higher.

### 3.3. Dilution

**Figure 8:**
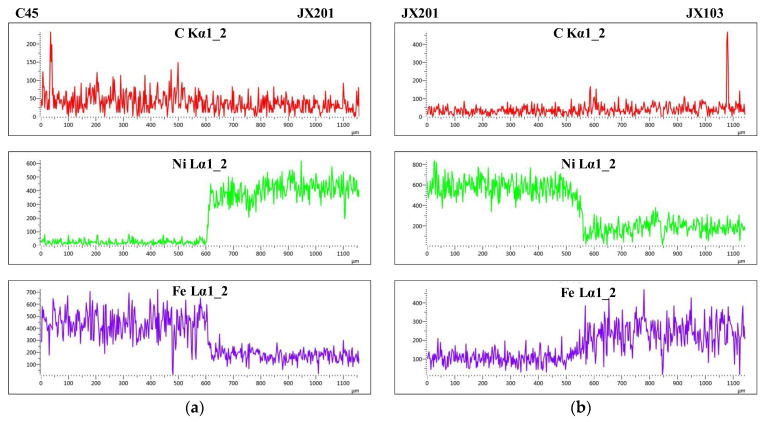
Element distributions of the two fusion boundaries: (**a**) Fusion boundary of JX201 and C45; (**b**) Fusion boundary of JX103 and JX201.

The dilution levels of the two fusion boundaries were compared by calculating the volumetric dilution (*Dv*) based on the weight percentage of Fe using Equation (1) [23] as follows:

(1) $$D_{V}={\left\{1+\frac{\rho_{s}}{\rho_{w}}\left(\frac{Fe_{s}-Fe_{c}}{Fe_{c}-Fe_{w}}\right)\right\}}^{-1}$$

where $\rho_{s}$ is the density of the substrate material, $\rho_{w}$ is the density of the welding material, $Fe_{s}$, $Fe_{c}$ and $Fe_{w}$ are the weight percentage of Fe in the substrate, the first-layer welded material, and the welding wire, respectively. The weight percentage of Fe in the substrate and the welded material were measured by the EDS. At each region, three lines were measured, and the average value was used for calculation. Table 2 shows the measured data and the calculated values. Both the dilution of JX201 to C45 and JX103 to JX201 are at a relatively low level (<30%), which implies a good mechanical property between the two welded layers [24]. Besides, the element distributions of C, Ni, and Fe in the mentioned two fusion boundaries were detected. The EDS results are shown in Figure 8. It can be found that the transition of Fe and Ni in the fusion boundary of JX103 and JX201 is smoother, and no decarburization phenomenon was found in either fusion boundary. 

### 3.4. Wear Behavior

**Figure 9:**
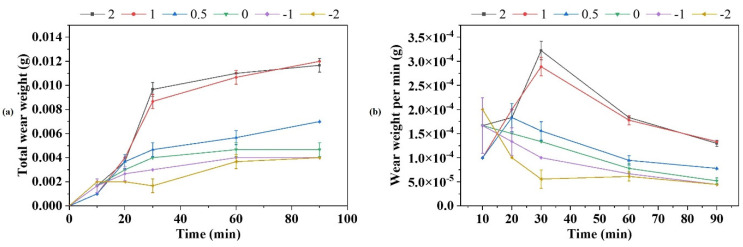
Wear weight comparison of the samples at the six positions shown in Figure 3 (3 samples at each position): (**a**) Total wear weight; (**b**) Wear weight per min.

The aim of the wear test is to investigate the influence of the last welded layer on the other surfacing welded layers. Figure 9 compares the total wear weight and the wear weight per minute of the 18 wear samples (six positions with three samples in each position), which remain at different thicknesses of JX103 on the surface. As can be seen in Figure 9a, the wear weight of all samples had the same trend: it increased sharply in the beginning and increased slower when the friction continued, which could result from the formation of smooth compacted oxide layers on the sliding surface [25]. Except that, the samples that remained JX103 on the surface (samples 2, 1, and 0.5) had a larger total wear weight than the samples without JX103. Since the wear resistance of JX201 is much higher than that of JX103, it is predictable that a JX103 welded layer would decrease the wear resistance of the surface. However, when the thickness of the JX103 decreased, the total wear weight decreased, which represents the increased wear resistance. When the thickness of the JX103 layer is 0.5 mm, the total wear weight tends to be close to the samples without the JX103 layer. The total wear weight was also different for those samples without the JX103 layer. Samples-1 and -2 had the same wear weight after 60 min, while sample 0 had a larger total wear weight. Combined with the microstructure of the fusion boundary, a possible explanation is that the residue of JX103 and its dilution decreased the wear resistance of JX201 to a certain level.

Furthermore, Figure 9b shows the wear weight per minute of the samples, in which the differences between the samples with JX103 and without can be more clearly presented. All the samples with JX103 had an obvious increasing trend in the beginning and then decreased after 30 min. The appearance of the peak point marked the change in the friction mechanism. In contrast, the curves of the samples without JX103 had no peak point. Their wear weight per minute kept decreasing throughout the whole experiment time. It is worth noting that the wear weight per 10 min of the sample with 0.5 mm JX103 was already very close to the samples without JX103. As for the samples without JX103 (samples-1 and -2), the wear weights showed slight differences (<0.001 g) when the sliding time was less than 30 min. This effect disappears when the sliding time is longer than 30 min. Therefore, the deposition of JX103 is slightly deleterious to the wear resistance of JX201.

**Figure 10:**
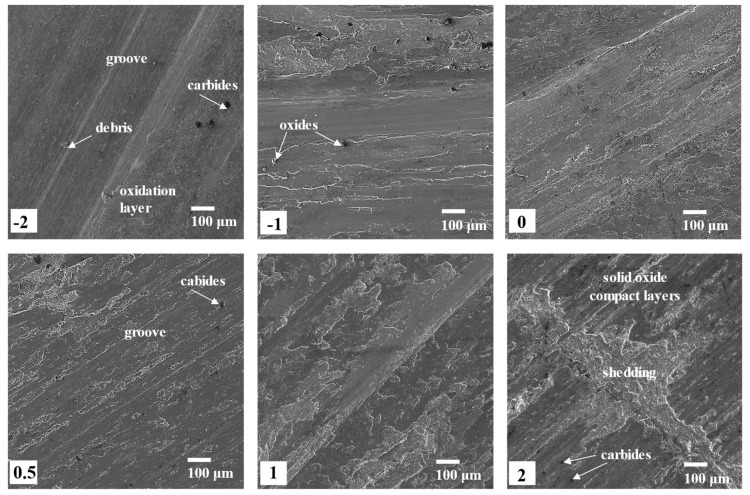
Worn surface morphology of the six series samples shown in Figure 3 (after 90 min).

After 90 min dry sliding wear, the morphologies of the worn surface of each sample are shown in Figure 10. As can be seen in these figures, the light region indicates the zone where its materials were removed from the surface, while the dark region illustrates the solid oxide compact layers, in which the materials remain but are denser than before due to the compression and oxidation of the sliding. Except for the solid oxide compact layers, grooves, shedding, carbides, and oxide particles were found on the worn surface of samples 2–0.5 [26,27], which indicates that both adhesive and abrasive wear happened during the sliding process. For these samples, as the thickness of the JX103 on the surface decreased, the area of the oxide compact layers and the shedding area obviously decreased. This can be related to the lower oxidation resistance of Fe-based superalloys compared to Ni-based superalloys. During the wear process, the JX103 was oxidized, and the oxide debris promoted three-body abrasive wear. The oxides have much higher hardnesses than the substrate materials, so they facilitate the formation of the grooves and plastic deformation of the substrate. Furthermore, together with the repeated loading and sliding, the oxides intensified the softening and oxidation of the material, finally leading to large flakes and debris peeling off [28]. Therefore, large shedding areas were observed. Severe abrasive and adhesive wear occurred on the surface of sample 2, but as the sample surface was closer to the fusion boundary, the severity of their wear decreased. This trend confirms the wear weight results in Figure 9. For sample-2, only a small number of debris and oxidation layers were observed except for the sliding grooves. Owing to the dispersed carbides and γ″-Ni3Nb phase, JX201 has a higher ability to withstand plastic deformation and adhesive wear. So the wear mechanism of sample-2 is mainly light abrasive wear. In conclusion, the difference between sample 2 and sample-2 indicates their different wear mechanisms, while the other samples represented the transition of these two wear mechanisms.

**Figure 11:**
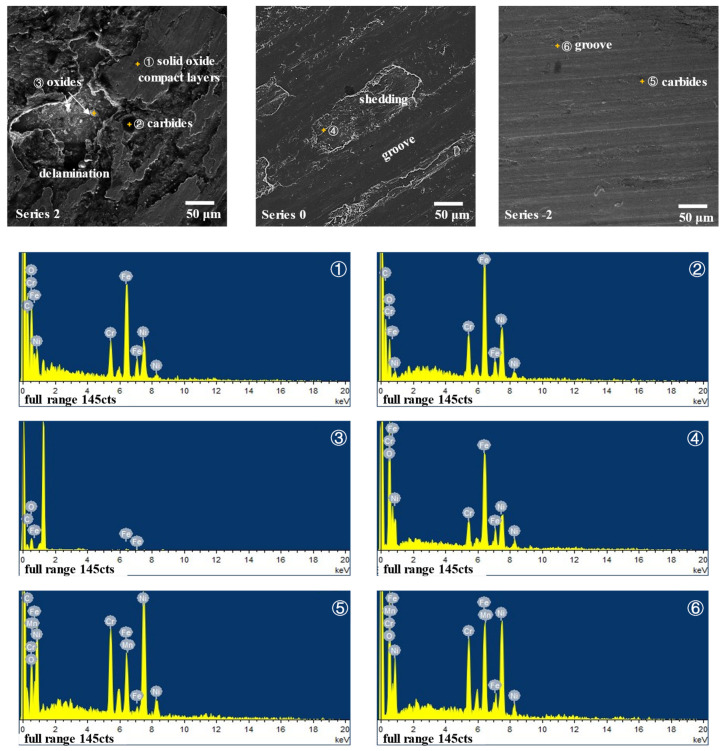
The typically worn surfaces of series 2, 0, and −2 and the EDS analysis of feature points.

Besides, the different carbides of the two materials also influence their wear mechanisms. As can be seen in Figure 5, before wear, JX103 contains a small number of carbides which are larger than those of JX201. Compared with the series of 0.5, 1, and 2 samples in Figure 10, it can be found that after 90 min of sliding, the surface contains even fewer carbides, and the carbides appear only at the solid oxide compact layers. A clearer comparison can be seen in Figure 11; series 2 (point ①, ②, ③) is the typically worn surface of JX103. Since the oxides of JX103 are porous and easily shed, the large carbides will easily fall off together with the oxides. The dislodged carbides aggravate the wear process, while in JX201 (point ⑤, ⑥), the carbides are small and dispersed, and the oxides are dense. The small and dispersed carbides enhance the wear resistance of the surface.

**Figure 12:**
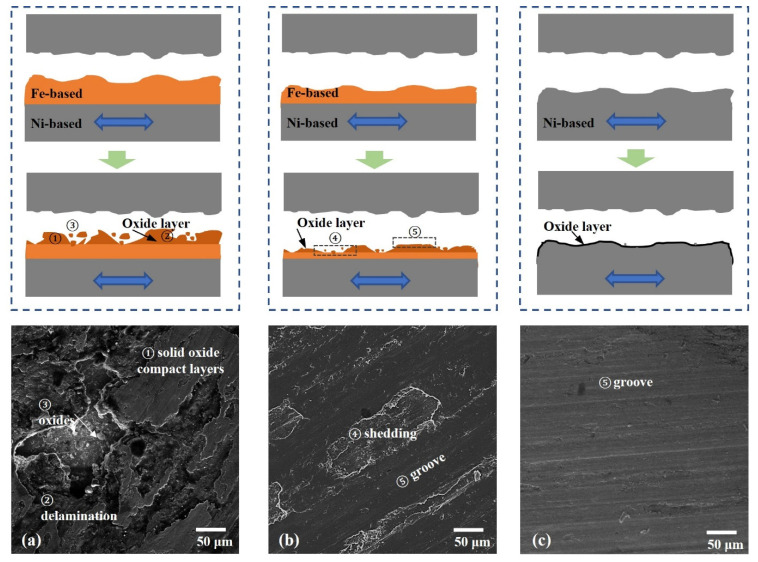
Wear mechanisms illustration. (**a**) series 2 ; (**b**) series 0 ; (**c**) series -2.

As illustrated in Figure 12, when the last welded layers changed from Ni-based to Fe-based material, the wear mechanism changed with the residual material amount. As we know, the Fe-based material, JX103, is more easily oxidized than the Ni-base material. When the surface consists only of Fe-based material, it will be easily oxidized, and its oxide film is mainly Fe_2_O_3_, which is porous and easily shed. Therefore, the thickness of the oxide film is great, and the shedding area on the surface is large. Significant delamination occurs on the wear surface, and lots of oxides and oxide layers can be seen here. The hard oxides intensify the process of oxidation and softening, which leads to a more severe wear process. Exfoliated oxides cause severe abrasive wear, and the intensified softening process causes severe adhesive wear. Differently, when the surface consists of only Ni-based material, after oxidation, it will form a dense oxide layer first and prevent further oxidation. The oxide layer is dense and not easy to peel off. Therefore only a small amount of oxides were found, and only light abrasive wear occurred. When the surface consists of both Fe-based material and Ni-based material, the oxide layer consists of both types of oxides, and the thickness of the oxide layer is therefore in between the other two scenarios. There are fewer oxides and a smaller shedding area. Both adhesive and abrasive wear occurs on the surface but not as severely as on surfaces consisting of only Fe-based material.

Overall, the wear test results indicate that by changing the material of the last welded layers from Ni- to Fe-based material, the wear mechanism will change with the residual amount of Fe-based material. The wear resistance decreases when residual Fe-based material increases. However, when the residual thickness is less than 0.5 mm, the wear weight per minute is already at the same level as the sample without Fe-based material. It indicates that changing the last welded layers could be an economically feasible approach to decrease the costs of the bimetallic layer surfacing method.

## 4. Conclusions

To improve machinability and reduce material waste of WAAM Ni-based superalloys, the influence of surfacing Fe-based alloy layers on WAAM Ni-based superalloys material on its microstructure and wear properties were investigated. The conclusions from this work are as follows:

(1) By changing the material of the last welded layers from Ni-based superalloy to Fe-based alloy, the mean surface hardness is increased from HV350 to HV400, and a smooth hardness transition was achieved after heat treatment.
(2) The tendency for the disappearance of γ-Fe and the transformation of the carbide form was found in the welded layers. Cellular dendritic growth and type-Ⅰ boundaries were observed in the fusion boundary between these two materials. However, no type-Ⅱ boundary occurred, indicating a good crack resistance of the fusion boundary.
(3) The dilution of the fusion boundary is at a relatively low level (<30%), and no decarburization phenomenon was found.
(4) The wear resistance of JX201 was decreased by changing the last layer to JX103; however, as the residual thickness of JX103 decreased, the influence gradually decreased. Meanwhile, the wear mechanism changed from severe abrasive and adhesive wear to light abrasive wear. When the thickness is less than 0.5 mm, the wear weight per minute is already at the same level as the sample without JX103.

## Figures and Tables

**Table 1:** Chemical composition of the materials (wt%).

Material	C	Mn	Cr	Si	Ni	Mo	Al	W	V	P	S	Fe	Nb
JX103	0.25	1.61	5.51	0.72	1.5	1.69	0.21	1.04	0.24	0.013	0.004		
JX201	0.02	2.8	19.5	0.5	67							2	2.5
C45	0.42~0.50	0.50~0.80	≤0.25	0.17~0.37	≤0.25	<0.10				≤0.035	≤0.035		

**Table 2:** Dilution calculation of the two boundaries.

	$\rho_{s}\;(g/{\mathrm{cm}}^{3})$	$\rho_{w}\;(g/{\mathrm{cm}}^{3})$	$Fe_{s}\;(\%)$				$Fe_{c}\;(\%)$				$Fe_{w}\;(\%)$	$\mathrm{Dilution}\;D_{V}\;(\%)$
JX201 to C45	7.8	8.3	92.1	93.2		92.7	8.7	13.5		2.8	2	7.36
			Avg.		92.7		Avg.		8.3			
JX103 to JX201	8.3	7.8	2.3	0		2.7	55.8	71.8		68.5	87	24.15
			Avg.		1.67		Avg.		65.4			

## Data Availability

Not applicable.
